# Cortico-Cortical White Matter Motor Pathway Microstructure Is Related to Psychomotor Retardation in Major Depressive Disorder

**DOI:** 10.1371/journal.pone.0052238

**Published:** 2012-12-20

**Authors:** Tobias Bracht, Andrea Federspiel, Susanne Schnell, Helge Horn, Oliver Höfle, Roland Wiest, Thomas Dierks, Werner Strik, Thomas J. Müller, Sebastian Walther

**Affiliations:** 1 University Hospital of Psychiatry, University of Bern, Bern, Switzerland; 2 Department of Psychiatric Neurophysiology, University of Bern, Bern, Switzerland; 3 Department of Diagnostic Radiology, Medical Physics, University Medical Centre Freiburg, Freiburg, Germany; 4 Departments of Radiology and Biomedical Engineering, Feinberg School of Medicine, Northwestern University, Chicago, Illinois, United States of America; 5 Institute of Diagnostic and Interventional Neuroradiology, University of Bern, Bern, Switzerland; University of South Florida, United States of America

## Abstract

Alterations of brain structure and function have been associated with psychomotor retardation in major depressive disorder (MDD). However, the association of motor behaviour and white matter integrity of motor pathways in MDD is unclear. The aim of the present study was to first investigate structural connectivity of white matter motor pathways in MDD. Second, we explore the relation of objectively measured motor activity and white matter integrity of motor pathways in MDD. Therefore, 21 patients with MDD and 21 healthy controls matched for age, gender, education and body mass index underwent diffusion tensor imaging and 24 hour actigraphy (measure of the activity level) the same day. Applying a probabilistic fibre tracking approach we extracted connection pathways between the dorsolateral prefrontal cortex (dlPFC), the rostral anterior cingulate cortex (rACC), the pre-supplementary motor area (pre-SMA), the SMA-proper, the primary motor cortex (M1), the caudate nucleus, the putamen, the pallidum and the thalamus. Patients had lower activity levels and demonstrated increased mean diffusivity (MD) in pathways linking left pre-SMA and SMA-proper, and right SMA-proper and M1. Exploratory analyses point to a positive association of activity level and mean-fractional anisotropy in the right rACC-pre-SMA connection in MDD. Only MDD patients with low activity levels had a negative linear association of activity level and mean-MD in the left dlPFC-pre-SMA connection. Our results point to structural alterations of cortico-cortical white matter motor pathways in MDD. Altered white matter organisation of rACC-pre-SMA and dlPFC-pre-SMA pathways may contribute to movement initiation in MDD.

## Introduction

Besides disturbances of mood and affect psychomotor retardation is a key feature of major depressive disorder (MDD) [1]. Psychomotor retardation involves speech, facial expression, posture, as well as pace and extent of movements [1], [2], [3]. Psychomotor slowing is of clinical relevance since it may help to distinguish depressive subtypes [1], [2], [4]. Furthermore, psychomotor slowing is associated with response to some antidepressants [1], [2], [3], [5]. Nevertheless, to date the neurobiology of psychomotor retardation in MDD is poorly understood.

Most findings of studies investigating associations of motor retardation and brain structure or function have been localised in prefrontal brain regions. For instance, in MDD associations of cerebral blood flow (CBF) and psychomotor slowing were found in the dorsolateral prefrontal cortex (dlPFC), the anterior cingulate cortex or the orbitofrontal cortex [6], [7], [8], [9], [10]. The rACC is involved in reinforcing behaviour and in anhedonia [11] whereas the dlPFC is essential for goal directed behaviour [12]. Neurobiological alterations of rACC and dlPFC may therefore contribute to prominent symptoms of MDD such as avolition and anhedonia and result in reduced daily activities [13]. In addition, psychomotor retardation was linked to CBF of the supplemental motor area [10] and to structural alterations and hypodopaminergic states of the basal ganglia [14], [15], [16]. White matter pathways connect these core regions of the motor system. Therefore, those cortico-cortical and cortico-basal ganglia connection pathways are of particular interest to understand psychomotor slowing in depression.

White matter microstructure reflects motor behaviour in healthy subjects. In fact, volitional motor activity, special motor skills and aerobic exercise are associated with white matter integrity [17], [18], [19], [20]. In addition, longitudinal studies demonstrated that training as well as limb immobilization may induce white matter changes of motor pathways [21], [22], [23]. Therefore, psychomotor retardation in MDD should be reflected by white matter changes of motor pathways as well.

Indeed, altered white matter microstructure has been demonstrated in MDD and healthy controls at risk for depression in major motor pathways such as the corpus callosum, thalamic projection fibres, the anterior limb of the internal capsule or the superior longitudinal fasciculus [24], [25], [26], [27], [28], [29]. However, an assessment of motor behaviour is required to link motor symptoms to underlying neurobiological alterations.

Actigraphy is a reliable measure of gross motor behaviour and repeatedly documented reduced motor activity in MDD [10], [30], [31]. Furthermore, actigraphy measures have been linked to symptom severity [32], [33]. Recently, our group used a voxel-based morphometry (VBM) based DTI approach in combination with actigraphy to relate motor activity to white matter integrity in MDD. Findings point to altered associations of white matter integrity and motor activity in white matter regions of the motor system located close to the dorsal pre-motor cortex and the primary motor cortex [34]. However, the connection pathways involved in these alterations are unclear.

Therefore, the present study was specifically designed to investigate pathways between core regions of the motor system such as the rostral anterior cingulate cortex (rACC), the dlPFC, the pre-supplementary motor area (pre-SMA), the SMA-proper, the M1 and the basal ganglia (thalamus, putamen, caudate nucleus, pallidum). We hypothesized that (i) psychomotor retardation is reflected by reduced volitional motor activity in MDD, (ii) structural connectivity between key areas of the motor system differs in MDD. Those alterations should be reflected by alterations of pathway connection probabilities as well as by reduced fractional anisotropy (FA) and increased mean diffusivity (MD) of the extracted pathways [35]. Furthermore, we performed exploratory analyses of motor pathways to investigate whether associations of quantitative motor behaviour and structural connectivity differ between MDD and healthy controls. Because psychomotor retardation was associated with prefrontal brain regions [6], [7], [8], [9], [10] and the particular importance of dlPFC and rACC for engaging in activities [12], [13] we expect alterations to be most pronounced in dlPFC-pre-SMA and rACC-pre-SMA pathways.

## Methods

### 2.1 Subjects

The sample includes subjects who participated in previous studies [10], [34]. We recruited 21 patients with MDD at the inpatient and outpatient departments of the University Hospital of Psychiatry, Bern, Switzerland and 21 healthy controls. All participants were right handed as assessed with the Edinburgh handedness inventory [36]. Before the scanning procedure participants were assessed with the Beck Depression Inventory (BDI) (controls = 1.6±2.1, patients = 29.9±8.8, Z = −5.58, p<0.001) [37], the Hamilton Depression Rating Scale (HAMD) (controls = 0.6±1.1, patients = 26.4±5.3, Z = −5.68, p<0.001) [38] and the Montgomery-Asberg Depression Rating Scale (MADRS) (controls = 0.9±1.4, patients = 26.8±4.9, Z = −5.61, p<0.001) [39]. Diagnoses were given according to DSM-IV following semi-structured clinical interviews. In the MDD group, 9 out of 21 patients had a history of more than 3 episodes. All patients were medicated at the time of scanning. All but two patients received antidepressant drugs (amitriptyline 25–225 mg, n = 2; escitalopram 20 mg, n = 2; clomipramine 75 mg, n = 1; doxepin 100–200 mg, n = 2; mirtazapine 15–45 mg, n = 4; sertraline 100–200 mg, n = 2; venlafaxine 150–300 mg, n = 2; and venlafaxine and mirtazapine combination 150–300 mg/30 mg, n = 4). Six patients received substances for augmentation (lithium n = 2; lamotrigine n = 1, quetiapine n = 3), and 7 patients received zolpidem 10 mg at night. We performed the structured clinical interview for DSM-IV part 2 (SCID-II) and the Unified Parkinson’s disease rating scale (UPDRS) [40] to exclude comorbid personality disorders or parkinsonian symptoms. Furthermore participants with a history of significant head trauma, electroconvulsive therapy, substance abuse or dependence other than nicotine were excluded from the study. Controls with a lifetime history of depressive episodes or first degree relatives with any affective disorder were excluded. The 21 healthy control subjects were matched for gender (controls = 42.9% male, patients = 52.4% male, Chi^2^ = 0.38, p = 0.758), age (controls = 41.0±13.7 years, patients = 45.0±13.7 years, T = −0.98, p = 0.332), years of education (controls = 14.7±4.3 years, patients = 13.3±2.6 years, T = 1.28, p = 0.211), body mass index (BMI) (controls = 23.6±3.9 kg/m^2^, patients = 25.1±5.1 kg/m^2^, T = −1.04, p = 0.303) and annual income (controls = 43743±22687 CHF, patients = 46101±31280 CHF, T = −0.28, p = 0.781). The study protocol was approved by the local ethics committee (KEK-BE 196/09) and was in accordance with the Declaration of Helsinki. All participants provided written informed consent.

### 2.2 Data Acquisition

#### 2.2.1. Actigraphy

We applied the same methods as in our previous work investigating motor behaviour in neuropsychiatric disorders e.g. [10], [41], [42]. After conduction of MRI scans which were performed between 8 and 10 am on Tuesdays and Wednesdays participants wore an actigraph (Actiwatch®, Cambridge Neurotechnology, Inc., UK) on the wrist of the left (nondominant) arm for 24 consecutive hours. Actigraphy of the non-dominant arm reflects total motor activity without interference of manual work [43]. Activity counts were stored in 2 s intervals. Participants provided sleep log information. Activity was analyzed exclusively during wake time. Data were read into a computer and extracted using Sleep analysis 5® software (Cambridge Neurotechnology, Inc., UK). The activity level (AL) (i.e. the cumulated activity counts during wake divided by the netto recording time in hours) was calculated with own Excel® templates [44].

#### 2.2.2. MRI acquisition

All images were acquired with a 12-channel signal reception head coil on a 3-Tesla MR scanner (Siemens Magnetom Trio, Erlangen, Germany). High-resolution T1-weighted MR images were obtained using a 3D Modified Driven Equilibrium Fourier Transform (MDEFT) sequence [45]. The optimized acquisition parameters were as follows: 176 sagittal slices, 256×224 matrix (with a non-cubic field of view (FOV) of 256 mm×224 mm, yielding a nominal isotropic resolution of 1 mm^3^), 7.92 ms repetition time (TR), 2.48 ms echo time (TE), 16° flip angle, inversion with symmetric timing (inversion time 910 ms), fat saturation and a12 min total acquisition time. Identical prescription of MR images was achieved using the Siemens Autoalign sequence, which automatically sets up consistent slice orientation based on a standard MRI atlas.

#### 2.2.3. Diffusion tensor imaging (DTI)

For DTI measurements, we used a spin-echo echo-planar-imaging (EPI) sequence (55 slices, FOV = 256×256 mm^2^, sampled on a 128×128 matrix resulting in 2 mm^3^ voxel size, TR/TE = 6000/78 ms) covering the whole brain (40 mT/m gradient, 5/8 partial Fourier, no acceleration factor, bandwidth 1346 Hz/Px). Diffusion-weighted images (DWI) were positioned in the axial plane parallel to the AC-PC line and measured along 42 directions with a b-value = 1300 s/mm^2^. Four DWI images were measured without diffusion weighting (i.e. b-value = 0, B0 image). These were scanned after the first diffusion-weighted image, and immediately after every 12^th^ subsequent diffusion-weighted image. We used a balanced and rotationally invariant diffusion-encoding scheme over the unit sphere to generate the DTI data [46].

### 2.3. Data Analysis

Imaging data were analysed using Statistical Parametric Mapping (SPM8) (www.fil.ion.ucl.ac.uk/spm), implemented in Matlab 7.6.0 (R2008a; Mathworks, Natick, MA, USA). We used a probabilistic fibre tracking method which allows for the extraction of pathways between two regions of interest (ROI) [47] (www.uniklinik-freiburg,de/mr/live/arbeitsgruppen/diffusion_en.html).

#### 2.3.1. ROIs

Regions of interest (ROIs) were selected using the WFU-Pick-Atlas implemented in SPM8 [48]. All ROIs were spatially located with regard to the Montreal Neurological Institute (MNI) reference brain. We chose the following bilateral ROIs: dlPFC, rACC, pre-SMA, SMA-proper, M1, thalamus, putamen, caudate nucleus and pallidum. The dlPFC is composed of Brodmann Area (BA) 9 and 46 [49]. We performed a vertical section through the genu of the ACC and only included parts of BA24 which were located rostral to the section for definition of the rACC [49], [50]. The border of the pre-SMA and the SMA-proper coincides with the vertical anterior commissural line of BA6 [51]. Therefore, we performed a vertical section through the anterior commissure in order to separate the BA6 into the two distinct ROIs.

#### 2.3.2. Pre-processing

The 4 B0 images were motion corrected using the SPM8 diffusion toolbox (http://sourceforge.net/projects/spmtools). DTI-images were then realigned to the B0 images. The T1-images were segmented into white and grey matter masks. This procedure calculates normalisation parameters for forward and backward transformation between individual native spaces and the MNI space. Because fibre tracking is done in the native space of each subject, all ROIs were transferred into the native space using the inverse normalisation parameters of each subject [52]. The T1-image and the inverse normalised ROIs of each subject were co-registered to an average image of the 4 corresponding B0-images.

#### 2.3.4. Probabilistic DTI-based fibre tracking

The applied probabilistic fibre tracking approach is described in depth in [47] and has been used in previous studies e.g. [52], [53], [54], [55], [56]. First, the diffusion tensor was computed [57] and second a Monte Carlo simulation of random walks similar to the probabilistic index of connectivity (Pico) method [58] was used to calculate probabilistic maps separately for each ROI. In these maps the visiting frequency of a voxel represents a degree of connectivity to the seed region. Random walks were limited to visit at maximum 150 voxels and were repeated 25000 times using Monte-Carlo simulations. The tracking area was restricted to the white matter masks obtained from the segmented T1-images to avoid tracking across anatomical borders. To ensure contact of the cortical ROIs with white matter, a rim of grey matter (10% of the grey matter mask) was included in the white-matter mask [53]. Second, region to region anatomical connectivity was computed using a combination of probability maps [47]. This requires a multiplication of the two resulting probability maps, which takes the directional information about the traversing trajectories into account in order to suppress merging and to preserve connecting fibres [47]. The values obtained from the combination of two probability maps represent a voxel-wise estimation of the **p**robability **i**ndex that a given voxel is part of the connecting fibre **b**undle of **i**nterest (PIBI).

By combining the probability maps, we bilaterally assessed structural connectivity between prefrontal-pre-SMA pathways (dlPFC - pre-SMA, rACC - pre-SMA), supplemental motor area-M1 pathways (pre-SMA - SMA-proper, SMA-proper - M1) and cortico-basal ganglia pathways (pre-SMA - caudate nucleus, pre-SMA - putamen, pre-SMA – pallidum, pre-SMA – thalamus, M1 - caudate nucleus, M1 - putamen, M1 - pallidum and M1– thalamus).

#### 2.3.5. Post-processing

The combined probability maps of each subject were scaled to between 0 and 1, spatially normalised into the standard MNI space, and smoothed using an isotropic 3-mm full width at half maximum Gaussian kernel.

#### 2.3.6. Statistical analysis

AL between groups was compared using independent t-tests. We aimed to investigate whether fibre tract organisation such as the course of pathways or pathway width differs between groups. Therefore, we used independent t-tests to compare PIBI values of normalised probability maps. T-tests were calculated in SPM8, using a threshold of p<0.05 with family-wise error (FWE) correction for multiple comparisons.

Furthermore, we compared white matter microstructure between groups by comparing the DTI based diffusion properties FA and MD. For each subject, native space ROIs were defined based on the combined probability maps. Only voxels that were considered to be part of a connection PIBI >0.0148 were included in these native space ROIs e.g. [53]. From the respective ROIs the diffusion properties mean-FA and mean-MD were extracted in order to compare white matter integrity. We calculated separated MANOVAS for 1) bilateral prefrontal-pre-SMA pathways (dlPFC-pre-SMA, rACC-pre-SMA) 2) bilateral supplemental motor area – M1 pathways (pre-SMA-SMA-proper, SMA-proper-M1) and 3) bilateral cortico-basal ganglia pathways (pre-SMA - caudate nucleus, pre-SMA - putamen, pre-SMA - pallidum and pre-SMA – thalamus, M1 - caudate nucleus, M1 - putamen, M1 - pallidum and M1– thalamus). Independent variable was group (controls vs. MDD) and dependent variables were mean-FA and mean-MD values extracted from the respective pathways.

Moreover, we investigated whether different pathways are involved in motor control in MDD. Therefore, we tested for differences between groups regarding the relationship of fibre tract organisation and quantitative motor behavior. For each of the 24 combined probability maps SPM8 was used to calculate a general linear model (GLM). Independent variable was group (controls vs. MDD) and dependent variables were PIBI and AL. A significance threshold of p<0.05, after FWE correction for multiple comparisons was applied.

In addition, we explored if controls and MDD patients differ regarding the relationship of quantitative motor behaviour and white matter integrity. Therefore, GLMs with the independent variable group (controls vs. MDD) and dependent variables mean-FA and AL respectively mean-MD and AL were computed for each of the 24 pathways. Those analyses were performed in SPSS 18 (Chicago, IL, USA). To date there is not sufficient data to generate a priori hypothesis whether or not specific pathways are affected. Therefore, the purpose of our analyses was the identification of candidate pathways and we set a level of significance of p<0.05. After identifying candidate pathways we used a Bonferroni correction for multiple comparisons (p<0.05/24 = 0.002).

Moreover, we performed additional analyses to strengthen our preliminary findings. We assumed that identified differences in associations of structural connectivity and quantitative motor behaviour are predominantly driven by MDD patients with more pronounced psychomotor retardation. Therefore, we generated a more and a less affected MDD group applying a median split of the AL-values. For those pathways where preliminary results revealed different associations of white matter integrity and motor behaviour, we calculated additional GLMs. Dependent variable was group (MDD with low AL, MDD with high AL, controls) and independent variables were AL and mean-FA (mean-MD respectively).

## Results

### 3.1. Group Comparisons of Activity Level

AL was higher in the control group (controls = 19599±7050, patients = 12417±6285, T = 3.485, df = 40, p = 0.001). AL was not correlated with age (r = −0.12, p = 0.453), duration of education (r = 0.07, p = 0.659), BMI (r = −0.15, p = 0.356) or mean depression scale scores (controls: HAMD r = 0.207, p = 0.368; BDI r = −0.084, p = 0.718; MADRS r = 0.012, p = 0.959; patients: HAMD r = −0.181, p = 0.434; BDI r = 0.073, p = 0.752; MADRS r = −0.027, p = 0.908). AL did not differ between antidepressant substance classes ((F(4, 21) = 1.088, p = 0.395).

### 3.2. Group Comparisons of Structural Connectivity of Motor Pathways

Independent t-tests of PIBI did not reveal differences between groups. Those t-tests were calculated in SPM8, p<0.05, FWE correction for multiple comparisons.

**Figure 1:**
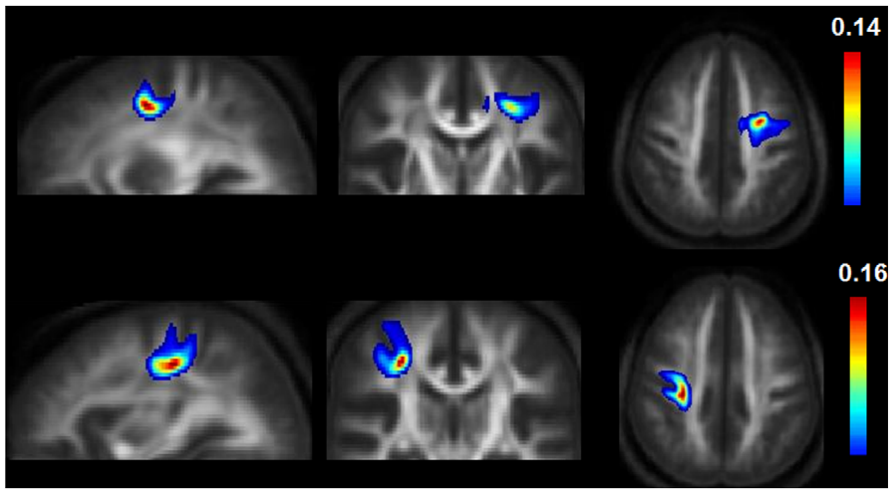
Supplemental motor area- primary motor cortex pathways with altered white matter microstructure. Mean maps averaged on both groups and overlaid on mean-FA images are displayed for the left pre-SMA-SMA-proper (first row) und the right SMA-proper-M1 (second row) connection. The voxel values represent an estimation of the probability that the voxel is part of the fibre bundle of interest (PIBI). To remove random artefacts, only voxels with PIBI values >0.0148 were included in probability maps [52], [53]. Maximum PIBI values are displayed at the top of each colour bar. The displayed figures illustrate anatomical pathways from where mean-MD values have been extracted.

Results of the six MANOVAs indicate white matter microstructure alterations of supplemental motor area – M1 connections (see Table 1 and Figure 1).

**Table 1:** MANOVAs of white matter microstructure.

Connections	F	df	p
**Prefrontal-pre-SMA (FA)**	0.529	4	0.715
**Prefrontal-pre-SMA (MD)**	0.470	4	0.757
**Supplemental motor area –M1 (FA)**	1.903	4	0.130
**Supplemental motor area –M1 (MD)**	3.051	4	0.029*
**Cortico-basal ganglia (FA)**	0.530	16	0.905
**Cortico-basal ganglia (MD)**	0.965	16	0.518

FA, mean-fractional anisotropy, MD, mean-mean diffusivity, M1 primary motor cortex, SMA supplementary motor area.

Calculating post-hoc independent t-tests for mean-MD of supplemental motor area-M1 pathways we found a significant increase of mean-MD in MDD in the left pre-SMA-SMA-proper (controls = 0.71±0.03, patients = 0.73±0.03, T = −2.478, df = 40, p = 0.018) as well as in the right SMA-proper-M1 connection (controls = 0.72±0.02, patients = 0.74±0.03, T = −2.7, df = 40, p = 0.011).

### 3.3. Associations of Activity Level and Structural Connectivity of Motor Pathways

**Figure 2:**
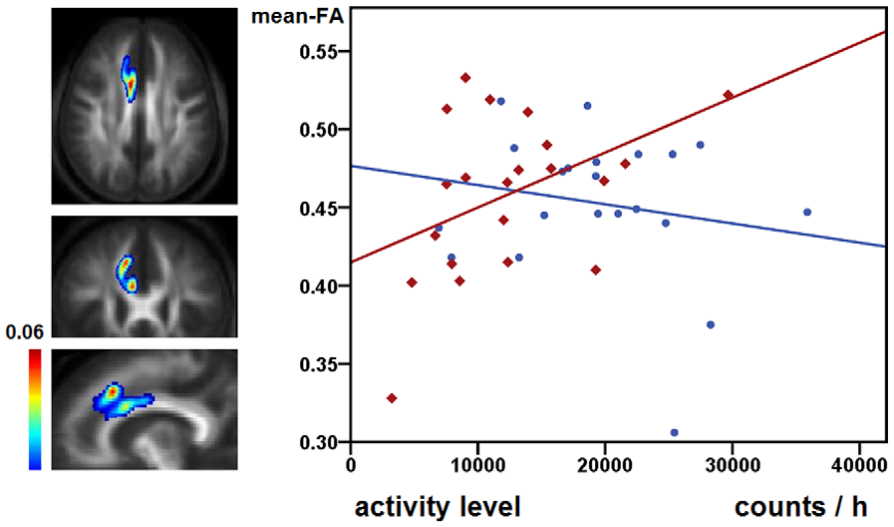
Association of white matter microstructure and activity level in the right rACC-pre-SMA pathway. The first column displays mean probability maps of the right rACC-pre-SMA connection averaged on both groups and overlaid on mean-FA images of the subjects. The voxel values represent an estimation of the probability that the voxel is part of the fibre bundle of interest (PIBI) To remove random artefacts, only voxels with PIBI values >0.0148 were included in probability maps [52], [53]. Maximum PIBI values are displayed at the top of the colour bar. The displayed figures illustrate anatomical pathways from where mean-FA values have been extracted. The second column displays mean-FA values extracted from the displayed connection and the corresponding activity level values of the respective subjects. Controls are displayed as blue circles, patients as red diamonds.

**Figure 3:**
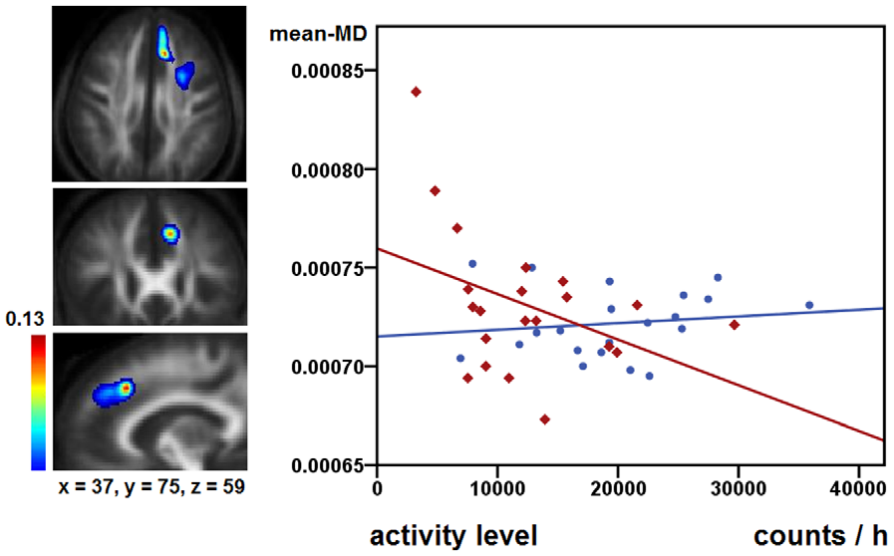
Association of white matter microstructure and activity level in the left dlPFC-pre-SMA pathway. The first column displays mean probability maps of the left dlPFC-pre-SMA connection averaged on both groups and overlaid on mean-FA images of the subjects. The voxel values represent an estimation of the probability that the voxel is part of the fibre bundle of interest (PIBI). To remove random artefacts, only voxels with PIBI values >0.0148 were included in probability maps [52], [53]. Maximum PIBI values are displayed at the top of the colour bar. The displayed figures illustrate anatomical pathways from where mean-MD values have been extracted. The second column displays mean-MD values extracted from the displayed connection and the corresponding activity level values of the respective subjects. Controls are displayed as blue circles, patients as red diamonds.

The GLMs for PIBI did not reveal significant group * AL interactions. Those analyses had been performed in SPM8, p<0.05, FWE correction for multiple comparisons. Furthermore, exploratory tests for associations with AL were performed for the extracted mean-FA and mean-MD values of the 24 pathways, two of them demonstrating significant group*AL interactions:

The GLM for mean-FA of the right rACC-pre-SMA connection indicated different group * AL interactions (F = 4.392, df = 1, p = 0.043), however, this result does not withstand Bonferroni correction (p<0.002). Patients but not controls had a significant positive correlation for mean-FA and AL in the right rACC-pre-SMA connection (controls r = −0.061, p = 0.792; patients r = 0.449, p = 0.041, see Figure 2). Furthermore, we found group * AL interactions for mean-MD for the left dlPFC-pre-SMA pathway (F = 4.292, df = 1, p = 0.045, see Figure 3), though, this result does not withstand Bonferroni correction (p<0.002). In both groups there were no significant correlations between mean-MD and AL (controls r = 0.230, p = 0.316; patients r = −0.340, p = 0.131).

Identified associations of AL and diffusion properties have been primarily driven by those MDD patients with more pronounced psychomotor retardation (see Figure S1 and Figure S2 in File S1). Neither were there significant correlations of depression scale scores and mean-FA of the right rACC-pre-SMA connection (HAMD r = −0.042, p = 0.793; BDI r = 0.035, p = 0.824, MADRS r = −0.112, p = 0.418) nor for depression scale scores and mean-MD of the left dlPFC-pre-SMA connection (HAMD r = 0.230, p = 0.316; BDI r = 0.239, p = 0.297; MADRS r = 0.397, p = 0.074).

## Discussion

The main finding of our study was reduced motor activity and altered structural connectivity of pre-SMA-SMA-proper and SMA-proper-M1 pathways in MDD. Furthermore, exploratory analyses point to an altered involvement of rACC-pre-SMA and dlPFC-pre-SMA pathways in motor control in MDD.

### 4.1. Group Comparison of Activity Level

As expected, AL was lower in MDD than in healthy controls. Our finding is in line with previous studies investigating psychomotor retardation with wrist actigraphy [10], [31], [34], [59]. At the time of the study all but two patients were on stable antidepressive medication. AL did not differ between types of antidepressant, use of mood stabilizers or zolpidem, as reported previously for this sample [34]. Therefore, we assume that medication did not systematically impact AL in the current sample. This is also in line with our previous study investigating 76 MDD patients [33]. Previous studies investigating the short term impact of different antidepressive drugs on AL yielded contradicting results [60], [61]. In either case, medication is used to treat psychopathology in MDD. Therefore in MDD patients with psychomotor retardation medication may have increased AL, whereas in MDD with anxious agitation AL may have decreased. For a detailed review of medication effects on activity levels we refer to the supplementary material of [34].

### 4.2. White Matter Integrity of Motor Pathways

We observed increased mean-MD in pre-SMA-SMA-proper and SMA-proper-M1 pathways in MDD. Pre-SMA and SMA-proper are essential for the initiation of movements, movement sequencing and motor learning [62], [63]. Therefore structural alterations of pre-SMA, SMA-proper and pathways linking these brain regions might be associated with motor symptoms in MDD. Indeed, previous studies reported volume reductions of the right pre-SMA in a group of patients with MDD who showed motor learning deficits [64]. Furthermore, grey matter volume reductions of the pre-SMA have been found in drug-naïve first episode MDD patients [65]. Pre-SMA, SMA-proper and M1 are interconnected via cortical association fibres as well as via parts of the superior longitudinal fasciculus (SLF) [66]. Our findings of increased mean-MD in MDD are in line with reductions of FA in the SLF in healthy controls at familial risk for depression as well as in MDD [25], [28], [29], [67]. Here, we extend previous work by specifically focussing on fibre tracts connecting pre-SMA, SMA-proper and M1 which are of particular importance for understanding motor behaviour.

Mean-FA and mean-MD did not differ between groups within dlPFC-pre-SMA and rACC-pre-SMA or within cortico-basal ganglia pathways. In contrast, previous studies reported decreased FA in MDD within the dlPFC [26], [68] as well as within the anterior limb of the internal capsule [24], [25]. However, particularly in cortico-basal ganglia circuits various pathways converge within the anterior limb of the internal capsule [69]. At present, the spatial resolution of DTI does not allow to disentangle pathways in areas of crossing fibres reliably [70], [71]. Therefore, involvement of other anatomical pathways might explain these discrepancies. Furthermore, one might speculate whether different clinical presentations of MDD might account for those inconsistencies. For instance, a previous study detected reductions of FA only in a subgroup of melancholic depressed patients but not when comparing MDD with healthy controls [26].

At present, only few studies applied DTI-based fibre tracking approaches to investigate MDD or patients at risk for depression [27], [72], [73], [74]. Our results of unchanged probability of connection which reflects the fibre tract organisation is in line with negative findings of [73] regarding the geometric characteristics of the cingulum bundle and the uncinate fasciculus in MDD. However, a previous study successfully classified patients with MDD from healthy controls with help of the number of fibres including motor pathways such as the corpus callosum and the cingulum bundle [74]. Therefore, further DTI fibre tracking studies are warranted to raise hypotheses on alterations of organisation of white matter pathways.

### 4.3. Association of Diffusion Properties and Activity Level

Our results point to altered associations of structural connectivity and motor activity in rACC-pre-SMA and dlPFC-pre-SMA pathways in MDD. Identified associations were more pronounced in patients with low activity level (see Figure S1 and Figure S2 in File S1).

Since neither mean-FA of the right rACC-pre-SMA nor mean-MD of the left dlPFC-pre-SMA connection or AL correlated with depression scale scores we assume an association which is distinct in MDD and specific for motor retardation but not for depression severity.

Functionally, the dlPFC is involved in initiation of motivated behaviour [75]. In MDD white matter microstructure abnormalities in the dlPFC have been reported pointing to neurobiological alterations [26], [68]. Moreover, in MDD CBF of the ACC was shown to correlate negatively with psychomotor retardation [6], [7]. Furthermore, the ACC is associated with action monitoring which has been shown to be impaired in MDD [76], [77], [78]. The present study identified impaired structural connectivity of pre-SMA-SMA-proper and SMA-proper-M1 pathways which are essential for volitional motor activity [62], [63]. One might speculate whether alterations of white matter microstructure of rACC -pre-SMA and dlPFC-pre-SMA pathways compensate for those structural and functional impairments in order to initiate movements. Furthermore, cortical engagement might substitute for basal ganglia dysfunction in MDD since reduced extracellular dopamine of caudate and putamen was reported in MDD with motor retardation [15], [16]. In our previous arterial spin labeling study we suggested a less pronounced impact of the inhibitory indirect pathway [79], [80] as compensation to maintain motor activity [10]. A more efficient use of rACC-pre-SMA and dlPFC-pre-SMA fibre tracts might foster movement initiation as well. This assumption is in line with a more pronounced involvement of further prefrontal brain regions such as the orbitofrontal cortex where we detected a positive association of CBF and motor activity in MDD which was not present in healthy controls [10].

In our previous VBM-based DTI-study MDD patients but not controls had a negative association of activity level and white matter integrity in a cluster localised underneath M1 [34]. Surprisingly, we could not detect such an association in fibre tracts emanating from the M1. This might be due to methodological differences. Whereas VBM-based DTI approaches aim at detecting subtle white matter pathologies, fibre tracking investigates whole fibre tracts. Likewise, a previous study detected white matter abnormalities with fibre tracking but not with TBSS investigating the same sample [72]. An alternative explanation might be that the identified cluster of our previous study [34] is not incorporated in the particular fibre tracts investigated in the present study.

### 4.4. Limitations

This study has some limitations. (i) Since all MDD patients were medicated we cannot rule out an impact of medication on AL. Nevertheless, in the current sample as well as in a larger sample [33] there was no effect of medication on AL (please refer to the supplementary material of [34]) (ii) Medication might have influenced white matter microstructure in MDD. However, findings on the effects of antidepressant drugs, benzodiazepines or antipsychotics on white matter microstructure in previous DTI studies and animal models are controversial [81], [82], [83]. (iii) The analyses of associations of white matter integrity of motor pathways and activity levels are truly exploratory and do not survive a Bonferroni correction for multiple comparisons. Therefore, identified associations may resemble false positives. However, we emphasize that the aim of this part of the study was to identify candidate pathways involved in psychomotor retardation in MDD. Our findings are reinforced because associations are driven by MDD patients with more pronounced psychomotor retardation (see Figure S1 and Figure S2 in File S1). Nevertheless, those findings must be interpreted with caution and require replication in future studies. (iv) DTI-based measures of FA and MD are unspecific regarding microstructural changes. Future studies should apply novel white matter mapping techniques to quantify axon density, diameter and myelination [84], [85].

### 4.5. Conclusions

Altered white matter integrity in motor pathways in MDD connecting pre-SMA, SMA-proper and M1 were identified in the present study. In MDD, dlPFC-pre-SMA and rACC-pre-SMA pathways might be used to initiate movements and to compensate for functional and structural alterations of cortical and subcortical motor regions. The findings suggest that pathways connecting motor cortices with the basal ganglia system do not seem to directly contribute to psychomotor retardation in MDD.

## Supporting Information

File S1 Exploratory analyses for associations of white matter integrity and activity levels for MDD patients with low AL and with high AL.
(DOC)
