# Supplementary material for: Cortico-Cortical White Matter Motor Pathway Microstructure Is Related to Psychomotor Retardation in Major Depressive Disorder
Source: PLoS One. 2012 Dec 20;7(12):e52238. doi: 10.1371/journal.pone.0052238 (PMC3527384; doi:10.1371/journal.pone.0052238)
Supplement: File S1 — Exploratory analyses for associations of white matter integrity and activity levels for MDD patients with low AL and with high AL. (DOC) [file pone.0052238.s001.doc]

The general linear model (GLM) revealed significant different interactions of activity level (AL) and mean-FA between MDD patients with low AL, MDD patients with high AL and controls for the right rACC-pre-SMA connection (F = 4.315, df = 2, p = 0.021). There were significant correlations of AL and mean-FA for patients with low AL (r = 0.618, p = 0.043) but not for patients with high AL (r = 0.406, p = 0.244) and for controls (r = -0.061, p = 0.792) (Figure S1).


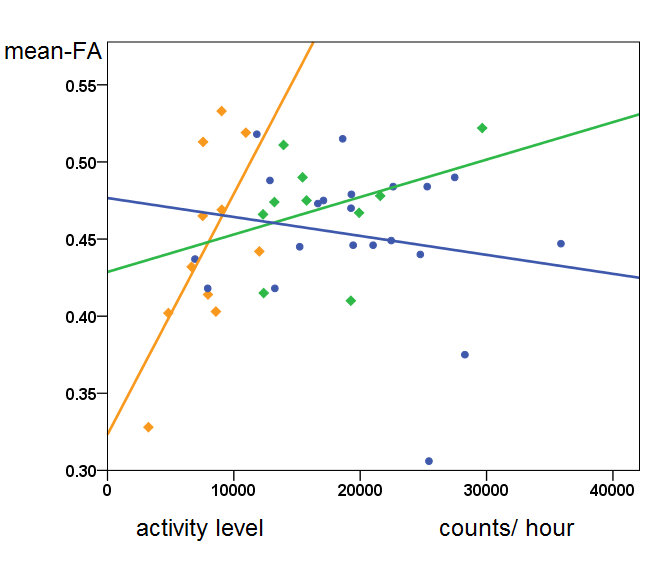


Figure S1. Mean-FA values extracted from the right rACC-pre-SMA connection and the corresponding activity level values of the respective subjects are displayed for MDD patients with low AL (orange diamonds), MDD patients with high AL (green diamonds) and for (blue circles).

A further general linear model (GLM) revealed significant different interactions of AL and mean-MD between MDD patients with low AL, MDD patients with high AL and controls for the left dlPFC-pre-SMA connection (F = 11.928, df = 2, p < 0.001). There were significant correlations of AL and mean-MD for patients with low AL (r = -0.629, p = 0.038) but not for patients with high AL (r = -0.286, p = 0.424) and for controls (r = -0.230, p = 0.316) (Figure S2).


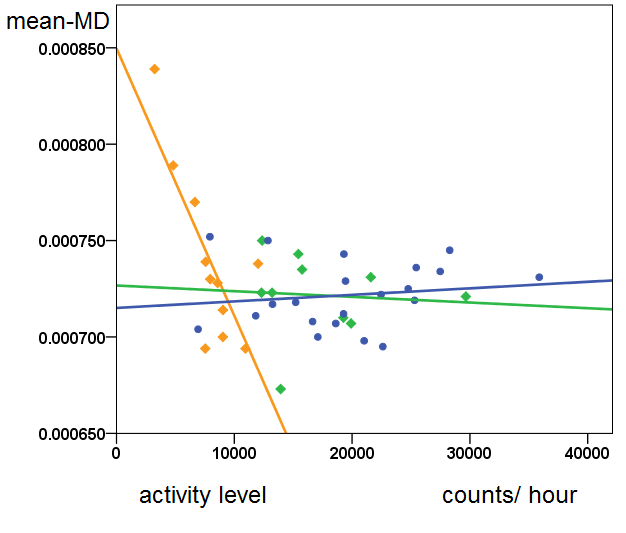


Figure S2. Mean-MD values extracted from the left dlPFC-pre-SMA connection and the corresponding activity level values of the respective subjects are displayed for MDD patients with low AL (orange diamonds), MDD patients with high AL (green diamonds) and for controls (blue circles).
